# Associations of Social Jetlag with Dietary Behavior, Physical Activity and Obesity among Chinese Adolescents

**DOI:** 10.3390/nu14030510

**Published:** 2022-01-25

**Authors:** Fang Liang, Jialin Fu, Yijia Xu, Yechuang Wang, Nan Qiu, Kai Ding, Jing Zeng, Justin B. Moore, Rui Li

**Affiliations:** 1 Department of Healthcare Management, School of Public Health, Wuhan University, Wuhan 430071, China; fliang@whu.edu.cn (F.L.); Fjl0708@whu.edu.cn (J.F.); seoyega@whu.edu.cn (Y.X.); ywang20@whu.edu.cn (Y.W.); 2013302170051@whu.edu.cn (N.Q.); 2021203050024@whu.edu.cn (K.D.); 2021283050065@whu.edu.cn (J.Z.); 2 Department of Implementation Science, Division of Public Health Sciences, Wake Forest School of Medicine, Winston-Salem, NC 27101, USA; jusmoore@wakehealth.edu; 3 School of Nursing, Wuhan University, Wuhan 430071, China

**Keywords:** social jetlag, body mass index, food consumption, physical activity, adolescent

## Abstract

This study aimed to investigate the associations between social jetlag (SJL), dietary behavior, physical activity, and weight status in Chinese youth. Data were derived from a cross-sectional survey in Wuhan, China in 2019. Information on SJL, the frequency of food and beverage consumption, physical activity, and BMI category were collected via a self-reported questionnaire. The Kruskal-Wallis test and ANOVA were conducted to determine differences in daily consumption frequency of food and beverage groups, BMI category, and physical activity among SJL groups. Logistic regressions and restricted cubic splines were performed to test the association between SJL and the incidence of overweight or obesity. A final sample of 3567 Chinese adolescents [mean (SD) age, 14.67 (1.72) years; 47.41% (1691) female] were included. Our findings demonstrated that adolescents with SJL may consume more unhealthy foods and fewer beneficial foods, while engaging in less moderate to vigorous physical activity (MVPA) and reporting higher BMIs. In addition, adolescents who experience more than 2 h of SJL had significant greater risk of overweight or obesity. Our findings on SJL of Chinese adolescents confirm the harmful effects of SJL and also provide insights into the etiology of obesity in Chinese adolescents.

## 1. Introduction

The proportion of overweight or obese children aged 5–19 years has increased rapidly in recent years such that in 2016 over 20% of the children in many countries were overweight or obese [1]. A growing body of research has indicated that obesity, which has long been considered a problem of high-income countries, is becoming more common in low- and middle-income ones as well [1,2,3]. By 2030, this proportion of youth with overweight or obesity is projected to exceed a quarter of the population in China [4]. It is worth noting that childhood obesity is widely known as a risk factor for early mortality and future morbidity, and preventing obesity has been regarded as the most promising strategy for the prevention of loss of life in modelling to 2040 [5,6].

Social jetlag (SJL) is defined as the difference in sleep-wake timing between weekdays and the weekend [7,8]. Due to the combination of both a shift towards later chronotype [9] and early school start times [10], adolescents experience short sleep during the school week and more SJL compared to adults [7]. Given the high prevalence of SJL among adolescents [11], increasing attention is being paid to evaluate its relationship with obesity among adolescents. However, results across the studies are paradoxical. Some studies found that SJL is associated with increased risk of obesity [12,13,14,15], and a study showed a strong positive association between SJL and Body Mass Index (BMI) [16], whereas another study demonstrated a negative relationship between SJL and BMI [17]. Also, recent research reported no association between SJL and BMI after adjustment for race/ethnicity [18]. Thus, more research needs to be conducted among adolescents. 

In addition, an unhealthy diet and physical inactivity are additional risk factors for obesity, and both of them are known to be linked to SJL. A previous study found that adolescents with more severe SJL consume more junk food and less fruits and vegetables [18]. Another recent cross-sectional study pointed out that SJL is related to a higher frequency of sugar-sweetened beverage consumption [13]. Also, a study indicated a positive association between SJL and increased moderate to vigorous physical activity (MVPA) [19]. Understanding how SJL relates to dietary behavior and physical activity may aid in the prevention of obesity. Furthermore, the relationship between SJL, dietary habits, physical activity and BMI have been shown to differ by race/ethnicity [18]. However, the majority of studies appear to have been focused on western cultures; to the best of our knowledge, no research has been conducted to date that investigates the association of SJL with dietary behavior, physical activity and weight status among Chinese adolescents. 

As SJL commonly occurs in Chinese adolescents [20], we designed the current study to evaluate how SJL is associated with dietary behavior, physical activity and weight status in Chinese adolescents. We hypothesized that adolescents with higher levels of SJL would consume more unhealthy foods and fewer beneficial foods, engage in less physical activity, report higher BMI, and display higher rates of overweight and obesity.

## 2. Methods

### 2.1. Ethics Statement

Ethical approval was granted by the Wuhan University Ethics Board (ethical approval code: 2019YF2056). Informed consent was obtained from all participants before enrollment. 

### 2.2. Participants

**Figure 1:**
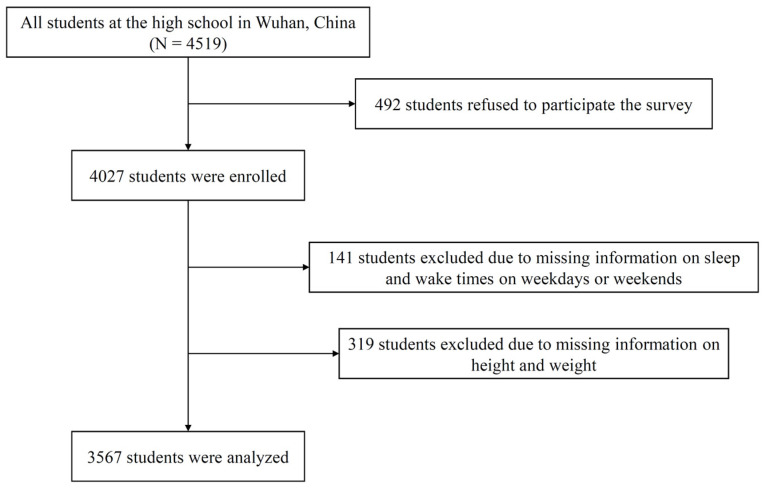
Flow chart of participants.

This school-based cross-sectional study was conducted in October 2019 among adolescents in a large high school in Wuhan, China. A total of 4519 adolescents at the high school were invited to participate in this study, and 89.11% of them consented to participate. Owing to the incomplete answers to the questionnaire (N = 460), the final analytic sample was composed of 3567 subjects (Figure 1). 

### 2.3. Measures

#### 2.3.1. Social Jetlag

SJL was determined by asking adolescents to report their usual bedtimes and wake times (hour: minute) on weekdays and weekends [21]. The midpoint of sleep was assessed by the sleep onset time and wake time. SJL was calculated by taking the absolute value of the product of subtracting the midpoint of sleep times on weekdays and weekends [7]. According to SJL status, we classified participants into three groups, namely no SJL (<1 h), mild SJL (1–2 h), and severe SJL (>2 h), as was done in previous studies [13,17,21]. 

#### 2.3.2. Frequency of Foods and Beverages Consumption

Questionnaires from the Family Life, Activity, Sun, Health, and Eating (FLASHE) Study, which was developed by the American National Cancer Institute, were used to collect data on dietary behavior [22]. Daily food intake frequency was examined using a self-report Dietary Screener Questionnaire which collects data on food items consumption during the past seven days, with frequency defined as 0 times/per week to three or more times per day [23]. Food and beverage data were converted to a daily frequency (e.g., one to three times in the past seven days = 0.29; four to s ix times in the past seven days = 0.71; one time per day = 1). The Dietary Screener Questionnaire is a validated questionnaire that was used for the National Health and Nutrition Examination Survey 2009–2010 [24]. The questionnaire was translated into Chinese and has been proven to have good reliability and validity [25,26]. We assessed reliability of the questionnaires using Cronbach’s alpha coefficient, and we performed exploratory factor analysis to evaluate validity: Cronbach’s Alpha = 0.86; Kaiser–Meyer–Olkin = 0.93, P Bartlett < 0.001. Some food and beverage groups were also presented in the original FLASHE data, such as junk foods, sugar sweetened beverages, fast foods, fruits and vegetables, all detrimental foods, and all beneficial foods [27]. More specific details of food and beverage consumption and food and beverage groups are presented in Supplementary Table S1.

#### 2.3.3. Physical Activity 

Physical activity was calculated as estimating minutes of MVPA according to the Youth Activity Profile (YAP) [28]. The YAP is a 15-item self-report instrument designed to assess physical activity level at school, during periods out of school, and on weekends for the previous week. Items in the “at school” section include a question asking how many days participants undertook active travel to and from school, and their activity levels during physical education, recess/study breaks, and lunch. Items in the “out of school” section included before school, after school, in the evening, and across both Saturday and Sunday. The YAP items were scored on a 5-point Likert scale, and scores of weekdays and weekends were averaged, respectively, to reflect the composite raw score for MVPA. Base on YAP raw scores, the time spent in MVPA was estimated using validated algorithms [29]. 

#### 2.3.4. Anthropometric Measurements

BMI was calculated with adolescents’ self-reported height and weight, while BMI Z-score values ((individual value − mean)/SD) were obtained following the criteria suggested by the World Health Organization (WHO) [30]. Overweight and obesity were classified according to the sex- and age-specific BMI reference values, which used as a standard as recommended by the Working Group on Obesity in China [31]. 

#### 2.3.5. Covariates

Demographic characteristics included age, gender, and monthly household income. Monthly household income was categorized as ≤5000 RMB, 5000–10,000 RMB, 10,000–20,000 RMB, 2000–40,000 RMB, or ≥40,000 RMB. Total sleep duration was defined as the average number of hours they slept per night and calculated from sleep duration on school nights and weekend nights [13]. 

### 2.4. Statistical Analysis

The results are presented as numbers (proportions) for categorical variables and as mean ± SD or median (IQR) for continuous variables depending on normality. The Kruskal-Wallis (KW) test with Bonferroni correction was performed to determine differences in daily consumption frequency of food and beverage groups and BMI among SJL groups. Different physical activity levels between SJL groups were analyzed with one-way ANOVA with Bonferroni tests. Logistic regression analyses were performed to test the association between SJL and overweight or obesity among Chinese adolescents. First, we only included SJL as the predictor. We then added demographic covariates (age, gender, and monthly household income). Lastly, we added total sleep duration and physical activity. We also conducted restricted cubic splines with three knots at the 10th, 50th, and 90th percentiles to flexibly model the association of SJL and the incidence of overweight or obesity. Likelihood ratio tests was used to test for potential non-linearity [32]. Statistical analyses were two-tailed, and *p*-values < 0.05 were used as the threshold for statistical significance, and were conducted using R software 4.0.2. 

## 3. Results

A total of 3567 adolescents were included in the analyses. Baseline characteristics of participants are presented in Table 1 according to categories of SJL. The mean (SD) age was 14.67 (1.72) years old. Among them, 1842 (51.64%) were male, and approximately 50% of their monthly household income were 5000–10,000 RMB. The mean (SD) total sleep time was 9.51 (3.72) hours, and the mean (SD) MVPA per day was 113.995 (28.76) minutes. 

As shown in Table 2, the daily frequency of consumption of junk foods, SSBs, fast foods, fruits and vegetables, all detrimental foods and all beneficial foods differed significantly among the 3 SJL groups (all KW *p* value < 0.01). Specifically, the daily frequency of consumption of junk foods, SSBs, fast foods, and all detrimental foods in the severe SJL group were higher than that of no SJL group (all Bonferroni-corrected *p* < 0.001). The daily frequency of fruits and vegetables (Bonferroni-corrected *p* = 0.006) and all detrimental foods (Bonferroni-corrected *p* = 0.001) in the severe SJL group were lower than that of no SJL group. 

Table 3 presents the mean time spent in MVPA by SJL groups. The minutes of MVPA for the total week (F = 5.600, *p* = 0.004), weekday out-of-school (F = 6.624, *p* = 0.001), and on weekends (F = 6.228, *p* = 0.002) differed significantly among the 3 SJL groups, while we found no significant differences in MVPA minutes for SJL groups for school-time (F = 2.881, *p* = 0.059). Specifically, the minutes of MVPA for the total week (Bonferroni-corrected *p* = 0.003), weekday out-of-school (Bonferroni-corrected *p* = 0.001), and on weekends (Bonferroni-corrected *p* = 0.003) were higher than that of the no SJL group. 

As shown in Table 4, the BMI differed significantly among the 3 SJL groups (KW = 9.896, *p* = 0.007), and the BMI in the severe SJL group was higher than that of the no SJL group (Bonferroni-corrected *p* = 0.025). The BMI Z score was not significantly different between the 3 SJL groups (KW = 0.659, *p* = 0.719). 

Table 5 shows the association of SJL with overweight or obesity. Relative to no SJL, after multivariable adjustment, participants having mild SJL showed no significant risk of overweight or obesity (adjusted OR = 0.97, 95% CI: 0.79–1.20, *p* = 0.787), whereas participants with severe SJL showed a significant increased risk of overweight or obesity (adjusted OR = 1.47, 95% CI: 1.14–1.91, *p* = 0.004). 

**Figure 2:**
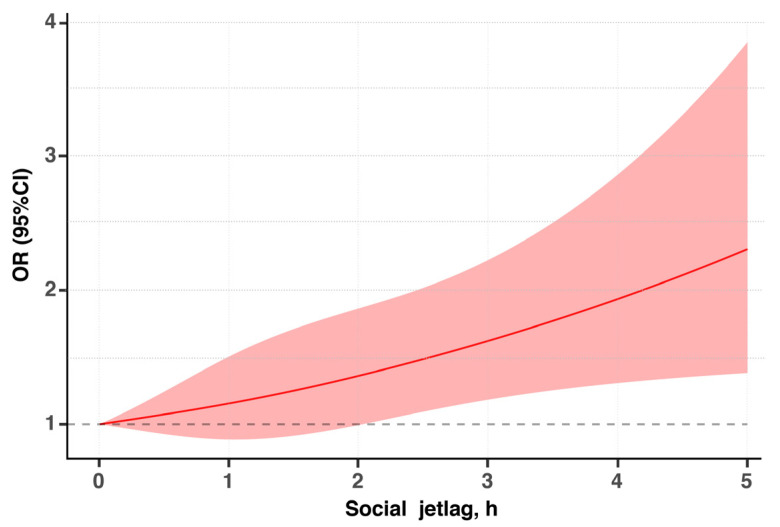
Association of social jetlag and overweight or obesity among Chinese adolescents. The solid lines indicate multivariate-adjusted odds ratios and the shaded areas indicate the 95% CIs. The reference point is 0 h for social jetlag, with knots located at the 10th, 50th, and 90th percentiles. The regression was adjusted for cofounders in Table 4.

In Figure 2, we used restricted cubic splines to flexibly model and visualize the relationship of SJL with overweight or obesity among Chinese adolescents. SJL (as a continuous variable) was linearly associated with risk of overweight or obesity, with a positive and monotonic association (*p* for non-linear trend = 0.867), and adolescents having more than 2 h SJL had a significant OR higher than 1.00. 

## 4. Discussion

The present study is the first research we know to explore the correlation between SJL, dietary behavior, physical activity and obesity in Chinese adolescents. We found that 54.64% of the adolescents were identified to have SJL, of which 17.07% of them were classified as severe SJL. Our findings demonstrated that adolescents with more severe SJL may consume more detrimental foods and fewer beneficial foods, while engaging in less MVPA, and report higher BMIs. In addition, adolescents who experience more than 2 h of SJL had a significantly greater risk of overweight or obesity. 

We found that 54.64% of the adolescents were identified to have SJL (≥1 h). A previous cross-sectional study of Chinese children that reported a relatively low proportion of SJL in the studied populations [20] and another study focused on Hispanic adolescents aged 8–16 years indicated that SJL occurred at the rate of 52.3% [19]. Besides, a prior American study that found that more than 80% of the adolescents had SJL [13]. Culture and geographical differences could be the reason for the different results between these studies, but further research is required to clarify this difference.

The results showed that adolescents with more severe SJL consume more unhealthy foods such as fast food, sugar sweetened beverages, and junk food, and less vegetables and fruits compared with no SJL. These results are consistent with findings from two prior epidemiological research studies conducted in America [13,18]. Past laboratory studies have shown that sleep patterns caused by changes in sleep-wake schedules can significantly affect appetite and satiety [33]. Likewise, epidemiologic studies are generally consistent in showing that sleep deprivation increases appetite and increases the desire for high-calorie foods [34,35]. Proposed mechanisms by which sleep deprivation may increase energy intake include more time and opportunities for eating, psychological distress, greater sensitivity to food reward, disinhibited eating, more energy needed to sustain extended wakefulness, and changes in appetite hormones [36]. 

The associations between SJL and physical activity are partially consistent with our hypothesis, and a previous cross-sectional study showed that adolescents with >2 h SJL were less physically active overall compared with those with ≤2 h SJL [37]. However, it is worth noting that there were significant differences in MVPA minutes between SJL groups at all other time periods except school time. A study suggested that recess and physical education classes are important moments for conducting MVPA because they contribute to 43% of the total physical activity [38]. In our study, the two key contributors to MVPA stayed the same during the school time between the three SJL groups. This may explain why MVPA in school time had a non-significant association with SJL. But further studies are certainly needed in order to obtain any conclusion. 

We found that adolescents of more severe SJL reported higher BMIs. In particular, an increased risk of overweight or obesity was found in those with SJL > 2 h. Relationships between SJL and BMI are in line with our hypothesis and some previous research in the field [12,13,14,15,16]. A recent cross-sectional study, conducted in 1556 American adolescents, pointed out that severe SJL is related to higher odds of having overweight or obesity compared with no SJL. Another cross-sectional study conducted in 804 American adolescents found that SJL is associated with greater adiposity in girls but not boys [12]. There are some potential biological mechanisms that may help explain the association. First, an experimental study found that short-term circadian misalignment resulted in a drop of leptin levels [39], which leads to increased food intake and reduced energy costs [40]. Second, another study suggested that reduced insulin sensitivity has been observed in people with circadian misalignment [41], and obesity is associated with reduced insulin sensitivity [42,43,44]. In contrast, a previous study on Chinese adults didn’t find any association between SJL and BMI [20]. Causes for the difference is not well understood and requires further elucidation.

This study has several limitations. First, the sample size of this research is relatively large, and participants in this study came from only one school, limiting the generalizability of the findings. Second, although we have adjusted a lot of confounding factors, the unmeasured factors may have an impact on the association between SJL, food consumption, physical activity and BMI. Third, the present study was based on the use of self-reported reported as opposed to objectively measured BMI. Forth, as the current study is a cross-sectional one, it restricted the ability to determine causality.

## 5. Conclusions

In conclusion, our study made an important contribution to the limited data on SJL of Chinese adolescents, and its effects on dietary behavior, physical activity and BMI. We demonstrated that adolescents with more severe SJL may suffer undesirable lifestyle habits, which are mainly manifested in eating more detrimental food, less beneficial food, being less physically active, and eventually resulted in higher BMIs. Additionally, adolescents who experience more than 2 h of SJL had a significantly greater risk of overweight or obesity. Our findings on SJL of Chinese adolescents confirm the harmful effects of SJL and also provide insights into the etiology of obesity in Chinese adolescents. Even so, more research is needed to clarify the specific pathways that connect SJL, dietary behavior, physical activity and obesity. Furthermore, a key aspect of subsequent research is motivating adolescents to build and maintain a regular sleep-wake cycle both on weekdays and on the weekend.

## Figures and Tables

**Table 1:** Baseline characteristics of participants.

Characteristic ^1^	Social Jetlag			Total (N = 3567)
	No (<1 h) (N = 1618)	Mild (1–2 h) (N = 1340)	Severe (>2 h) (N = 609)	
Age, years	14.65 (1.69)	14.65 (1.76)	14.73 (1.74)	14.67 (1.72)
Gender ^2^				
Male	881 (54.45%)	669 (49.93%)	292 (47.95%)	1842 (51.64%)
Female	721 (44.56%)	661 (49.33%)	309 (50.74%)	1691 (47.41%)
Missing	16 (0.99%)	10 (0.74%)	8 (1.31%)	34 (0.95%)
Monthly household income, RMB ^2^				
≤5000	211 (13.04%)	181 (13.51%)	75 (12.32%)	467 (13.09%)
5000–10,000	758 (46.85%)	622 (46.42%)	262 (43.02%)	1642 (46.03%)
10,000–20,000	389 (24.04%)	326 (24.33%)	151 (24.79%)	866 (24.28%)
2000–40,000	88 (5.44%)	80 (5.97%)	43 (7.06%)	211 (5.92%)
≥40,000	46 (2.84%)	31 (2.31%)	34 (5.58%)	111 (3.11%)
Missing	126 (7.79%)	100 (7.46%)	44 (7.22%)	270 (7.57%)
Overweight or obesity ^2^	290 (17.92%)	226 (16.87%)	133 (21.84%)	649 (18.20%)
Total sleep time, h	9.03 (3.45)	9.84 (3.90)	10.05 (3.82)	9.51 (3.72)
Social jetlag, h	0.47 (0.29)	1.41 (0.31)	2.83 (0.78)	1.23 (0.94)

^1^ Values are mean (SD), unless otherwise specified. ^2^ Values are n (%).

**Table 2:** The differences in daily consumption frequency of food and beverage groups among social jetlag groups.

Food and Beverage Groups	No SJL (<1 h)		Mild SJL (1–2 h)		Severe SJL (>2 h)		*p* Value
	Median (IQR)	Mean Rank	Median (IQR)	Mean Rank	Median (IQR)	Mean Rank	
Junk foods ^1^	1.13 (1.00)	1720.00	1.13 (1.00)	1764.01	1.42 (1.29)	1998.01	<0.001
SSBs ^2^	1.00 (0.58)	1688.21	1.00 (0.58)	1777.95	1.42 (1.00)	2051.80	<0.001
Fast foods ^3^	1.00 (0.87)	1726.62	1.13 (0.87)	1764.43	1.16 (1.00)	1979.51	<0.001
Fruits and vegetables ^4^	2.87 (2.42)	1832.45	2.71 (2.58)	1772.02	2.71 (2.58)	1681.64	0.008
All detrimental foods ^5^	3.03 (2.87)	1695.27	3.16 (3.13)	1765.09	4.00 (4.00)	2061.35	<0.001
All beneficial foods ^6^	4.58 (3.00)	1843.32	4.45 (3.13)	1768.47	4.16 (3.42)	1660.57	0.001

SJL: social jetlag; IQR: interquartile range; SSBs: sugar sweetened beverages. ^1^ Junk foods include the frequency of consumption of potato chip, candy/chocolate, cookie/cake, fried potato, and frozen dessert. ^2^ Sugar sweetened beverages include the frequency of consumption of soda, energy drink, sweetened fruit drink and sport drink. ^3^ Fast foods include the frequency of consumption of fried potato, fried chicken, pizza, taco, burger, and heat-and-serve food. ^4^ Fruits and vegetables include the frequency of consumption of 100% fruit juice, fruit, green salad, other nonfried vegetables, cooked beans, and other potatoes consumption frequency. ^5^ All detrimental foods include the frequency of consumption of pizza, heat-and-serve food, tacos, fried chicken, burger, processed meat, fried potatoes, candy/chocolate, cookies/cake, potato chips, frozen dessert, sugary cereal, sweetened fruit drink, soda, energy drink and sport drinks. ^6^ All beneficial foods include the frequency of consumption of 100% fruit juice, water, fruit, green salad, other nonfried vegetable, cooked beans, whole-grain bread, cooked whole grains, no sugary cereal and other potatoes consumption frequency.

**Table 3:** The differences in physical activity among social jetlag groups.

MVPA Per Week (min)	No SJL (<1 h)	Mild SJL (1–2 h)	Severe SJL (>2 h)	*p* Value
Total weekly	817.03 (169.36)	807.83 (181.23)	788.33 (203.39)	0.004
School-time	338.62 (80.90)	337.80 (85.65)	328.80 (92.43)	0.059
Weekday out-of-school	307.12 (66.81)	302.61 (71.36)	294.89 (80.19)	0.001
Weekend	171.31 (39.09)	167.78 (42.61)	164.65 (46.95)	0.002

MVPA: moderate to vigorous physical activity; SJL: social jetlag.

**Table 4:** The differences in BMI among social jetlag groups.

Variable	No SJL (<1 h)		Mild SJL (1–2 h)		Severe SJL (>2 h)		*p* Value
	Median (IQR)	Mean Rank	Median (IQR)	Mean Rank	Median (IQR)	Mean Rank	
BMI	19.71 (4.19)	1587.71	19.49 (4.15)	1563.21	20.02 (4.89)	1710.58	0.007
BMI Z score	0 (1.38)	1779.53	0 (1.52)	1797.47	0 (1.49)	1757.46	0.719

SJL: social jetlag; IQR: interquartile range; BMI: body mass index.

**Table 5:** The association between social jetlag and overweight or obese among Chinese adolescents.

	Unadjusted Model		Adjusted Model 1 ^a^		Adjusted Model 2 ^b^	
	OR (95% CI)	*p* Value	OR (95% CI)	*p* Value	OR (95% CI)	*p* Value
No SJL (<1 h)	1.00 (ref.)		1.00 (ref.)		1.00 (ref.)	
Mild SJL (1–2 h)	0.92 (0.75–1.13)	0.431	0.96 (0.78–1.18)	0.680	0.97 (0.79–1.20)	0.787
Severe SJL (>2 h)	1.32 (1.04–1.70)	0.025	1.41 (1.10–1.81)	0.007	1.47 (1.14–1.91)	0.004

OR: Odds ratio; SJL: Social jetlag. ^a^ age, sex, and monthly household income were adjusted. ^b^ physical activity level, intake of all beneficial foods, intake of all detrimental foods and total sleep duration were additionally adjusted.

## Data Availability

The data presented in this study are available on request from the corresponding author. The data are not publicly available due to privacy restrictions.

## Supplementary Materials

The following supporting information can be downloaded at: https://www.mdpi.com/article/10.3390/nu14030510/s1, Table S1. Measures of frequency of foods and beverages consumption.
