# Supplementary material for: Associations of Social Jetlag with Dietary Behavior, Physical Activity and Obesity among Chinese Adolescents
Source: Nutrients. 2022 Jan 25;14(3):510. doi: 10.3390/nu14030510 (PMC8840712; doi:10.3390/nu14030510)
Supplement: Supplementary file 1 [file nutrients-14-00510-s001.zip › nutrients-1552043-supplementary.pdf]

Table S1. Measures of frequency of foods and beverages consumption.

	Items	Response options	Scoring
**Junk foods** [1]	During the past 7 days, how many times did you eat regular potato chips?<br>During the past 7 days, how many times did you eat any type of candy or chocolate?<br>During the past 7 days, how many times did you eat cookies or cakes?<br>During the past 7 days, how many times did you eat fried potatoes?<br>During the past 7 days, how many times did you eat ice cream or other frozen desserts?	Never = 0,<br>1 – 3 times in the past 7 days = 0.29,<br>4 – 6 times in the past 7 days = 0.71,<br>1 time per day = 1,<br>2 times per day = 2,<br>3 or more times per day = 3.	Total score
**Sugar sweetened beverages** [2]	During the past 7 days, how many times did you drink regular soda?<br>During the past 7 days, how many times did you drink energy drinks?<br>During the past 7 days, how many times did you drink sweetened fruit drinks?<br>During the past 7 days, how many times did you drink sports drinks?	Never = 0,<br>1 – 3 times in the past 7 days = 0.29,<br>4 – 6 times in the past 7 days = 0.71,<br>1 time per day = 1,<br>2 times per day = 2,<br>3 or more times per day = 3.	Total score
**Fast foods** [3]	During the past 7 days, how many times did you eat fried potatoes?<br>During the past 7 days, how many times did you eat fried chicken?<br>During the past 7 days, how many times did you eat pizza?<br>During the past 7 days, how many times did you eat tacos?<br>During the past 7 days, how many times did you eat hamburgers or cheeseburgers?<br>During the past 7 days, how many times did you eat foods that you heat and serve or make from a box?	Never = 0,<br>1 – 3 times in the past 7 days = 0.29,<br>4 – 6 times in the past 7 days = 0.71,<br>1 time per day = 1,<br>2 times per day = 2,<br>3 or more times per day = 3.	Total score

**Fruits and vegetables** [4]	During the past 7 days, how many times did you drink 100% pure fruit?<br>During the past 7 days, how many times did you eat fruit?<br>During the past 7 days, how many times did you eat a green salad?<br>During the past 7 days, how many times did you eat other non-fried vegetables?<br>During the past 7 days, how many times did you eat other cooked beans?<br>During the past 7 days, how many times did you eat any other kind of potatoes?	Never = 0,<br>1 – 3 times in the past 7 days = 0.29,<br>4 – 6 times in the past 7 days = 0.71,<br>1 time per day = 1,<br>2 times per day = 2,<br>3 or more times per day = 3.	Total score
**All detrimental foods** [5]	During the past 7 days, how many times did you eat pizza?<br>During the past 7 days, how many times did you eat foods that you heat and serve or make from a box?<br>During the past 7 days, how many times did you eat tacos?<br>During the past 7 days, how many times did you eat fried chicken?<br>During the past 7 days, how many times did you eat hamburgers or cheeseburgers?<br>During the past 7 days, how many times did you eat processed meat?<br>During the past 7 days, how many times did you eat fried potatoes?<br>During the past 7 days, how many times did you eat any type of candy or chocolate?<br>During the past 7 days, how many times did you eat cookies or cakes?<br>During the past 7 days, how many times did you eat regular potato chips?<br>During the past 7 days, how many times did you eat ice cream or other frozen desserts?<br>During the past 7 days, how many times did you eat sugary cereals?<br>During the past 7 days, how many times did you drink sweetened fruit drinks?<br>During the past 7 days, how many times did you drink regular soda?<br>During the past 7 days, how many times did you drink energy drinks?<br>During the past 7 days, how many times did you drink sports drinks?	Never = 0,<br>1 – 3 times in the past 7 days = 0.29,<br>4 – 6 times in the past 7 days = 0.71,<br>1 time per day = 1,<br>2 times per day = 2,<br>3 or more times per day = 3.	Total score

**All beneficial foods** [6]	During the past 7 days, how many times did you drink 100% pure fruit?<br>During the past 7 days, how many times did you drink any water?<br>During the past 7 days, how many times did you eat fruit?<br>During the past 7 days, how many times did you eat a green salad?<br>During the past 7 days, how many times did you eat other non-fried vegetables?<br>During the past 7 days, how many times did you eat other cooked beans?<br>During the past 7 days, how many times did you eat whole grain bread?<br>During the past 7 days, how many times did you eat other cooked whole grains?<br>During the past 7 days, how many times did you eat non-sugary cereals?<br>During the past 7 days, how many times did you eat any other kind of potatoes?	Never = 0,<br>1 – 3 times in the past 7 days = 0.29,<br>4 – 6 times in the past 7 days = 0.71,<br>1 time per day = 1,<br>2 times per day = 2,<br>3 or more times per day = 3.	Total score

Note: [1]Junk foods include the frequency of consumption of potato chip, candy/chocolate, cookie/cake, fried potato, and frozen dessert. [2]Sugar sweetened beverages include the frequency of consumption of soda, energy drink, sweetened fruit drink and sport drink. [3]Fast foods include the frequency of consumption of fried potato, fried chicken, pizza, taco, burger, and heat-and-serve food. [4]Fruits and vegetables include the frequency of consumption of 100% fruit juice, fruit, green salad, other nonfried vegetables, cooked beans, and other potatoes consumption frequency. [5]All detrimental foods include the frequency of consumption of pizza, heat-and-serve food, tacos, fried chicken, burger, processed meat, fried potatoes, candy/chocolate, cookies/cake, potato chips, frozen dessert, sugary cereal, sweetened fruit drink, soda, energy drink and sport drinks. [6]All beneficial foods include the frequency of consumption of 100% fruit juice, water, fruit, green salad, other nonfried vegetable, cooked beans, whole-grain bread, cooked whole grains, no sugary cereal and other potatoes consumption frequency.
